# Metabolic Syndrome and Its Components Are Associated With Altered Amino Acid Profile in Chinese Han Population

**DOI:** 10.3389/fendo.2021.795044

**Published:** 2022-01-04

**Authors:** Shuiya Sun, Dongjuan He, Cheng Luo, Xihua Lin, Jiahua Wu, Xueyao Yin, Chengfang Jia, Qianqian Pan, Xuehong Dong, Fenping Zheng, Hong Li, Jiaqiang Zhou

**Affiliations:** ^1^ Department of Endocrinology, Sir Run Run Shaw Hospital, Zhejiang University School of Medicine, Hangzhou, China; ^2^ Department of Endocrinology, People’s Hospital of Quzhou, Quzhou, China

**Keywords:** amino acid, metabolic syndrome, component, biomarkers, amino acid profile

## Abstract

**Objective:**

Recent studies have found that the levels of plasma amino acids, such as branched-chain amino acids and aromatic amino acids, were associated with visceral obesity, insulin resistance, future development of diabetes and cardiovascular diseases. However, few studies have involved a Chinese Han population. This study aimed to examine the association between amino acid profile and metabolic syndrome (MetS) and its components in the Chinese Han population.

**Methods:**

This is a cross-sectional study, which enrolled a cohort of 473 participants from a community. We employed the isotope internal standard method to determine the plasma concentrations of 28 amino acids using high-performance liquid chromatography-tandem mass spectrometry (LC/MS). Participants were divided into MetS (*n* = 72) and non-MetS groups (*n* = 401) to analyze the association between amino acids and MetS and its components.

**Results:**

The prevalence of MetS was 15.2% according to the criteria. Plasma concentrations of isoleucine (Ile), leucine (Leu), valine (Val), tyrosine (Tyr), tryptophan (Trp), phenylalanine (Phe), glutamic acid (Glu), aspartic acid (Asp), alanine (Ala), histidine (His), methionine (Met), asparagine (Asn), and proline (Pro) were significantly higher in the MetS group than those in the non-MetS group (*P* < 0.05), but taurine (Tau) was significantly lower (*P* < 0.05). When MetS components were increased, the concentrations of these 13 amino acids significantly increased (*P* < 0.05), but Tau concentration was significantly decreased (*P* < 0.05). We extracted the amino acid profile by principal component analysis (PCA), PC1 and PC2, which extracted from the 14 amino acids, were significantly associated with MetS (odds ratio, 95% confidence interval: 1.723, 1.325–2.085 and 1.325, 1.043–1.684, respectively). A total of 260 non-MetS participants were followed up effectively, and 42 participants developed new-onset MetS within 5 years. We found that the amino acid profile of PC1 was linked to the occurrence of future MetS. Decreased Tau was correlated with the future development of MetS.

**Conclusion:**

Participants with MetS exhibit an abnormal amino acid profile, and its components gradually increase when these amino acids are altered. Amino acid PCA profile can be employed for assessing and monitoring MetS risk. Finally, decreased Tau may be linked to the future development of MetS.

## Introduction

Rapid lifestyle and dietary changes have contributed to a rise in the global prevalence of metabolic syndrome (MetS). MetS is a cluster of risk factors that increase the risk of an individual developing heart disease, diabetes, stroke, and chronic neurodegenerative disease (1–4). MetS diagnosis increases the relative risk for cardiovascular disease over 5 to 10 years by approximately 2-fold and type 2 diabetes mellitus (T2DM) by at least 5-fold (5). Current research on MetS pathogenesis mainly focuses on abdominal obesity, lipotoxicity, and insulin resistance. Recent studies have demonstrated that insulin resistance is strongly linked to amino acid metabolism, and it is believed that plasma amino acid levels may increase during insulin resistance. Branched-chain and aromatic amino acids (BCAAs and AAAs), in particular, are closely associated with the risk of visceral obesity, insulin resistance, and development of DM in the future. Plasma amino acid alterations in the early stage of lifestyle-related diseases are due to obesity and insulin resistance-related inflammation, and these alterations are reversed by appropriate (nutritional, pharmaceutical, or surgical) interventions that improve insulin sensitivity (6–10).

Accumulating evidence suggests that some amino acids could regulate various metabolic processes, including glucose and lipid metabolism. Evidence from American, Northern European, and Japanese populations link BCAAs and AAAs to insulin resistance, T2DM, and cardiometabolic risk. Research has demonstrated that the BCAAs and AAAs are positively correlated with body mass index (BMI), waist circumference, visceral fat, systolic blood pressure (SBP), diastolic blood pressure (DBP), fasting blood glucose, insulin and triglyceride levels, and insulin resistance, but inversely linked to high-density lipoprotein-cholesterol (HDL-c) in cross-sectional analyses of large prospective cohort studies (11–15). Interactions of excess BCAAs and lipids may cause β-cell dysfunction, accelerating the transition from an obese, insulin-resistant state to T2DM (16). Numerous studies have demonstrated that humans possess amino acid sensors that detect changes in amino acid levels and trigger corresponding metabolic responses, such as those mediated by serine/threonine-protein kinase general control non-derepressible 2 (GCN2), activating transcription factor 4 (ATF4), mechanistic target of rapamycin (mTOR), and 5′-adenosine monophosphate-activated protein kinase (AMPK) (17–19). Tremblay and Marette (20) demonstrated that BCAAs could downregulate glucose transport *via* the mTOR kinase pathway in skeletal muscle cells. Although little is known about the mechanisms underlying the increase in AAAs, it has been hypothesized that Tyr aminotransferase is repressed during states of insulin resistance and DM, resulting in elevations of circulating Tyr and Phe (21). Glycine (Gly) levels are lower among obese subjects compared with those with normal weight (22), and Gly has been inversely associated with BMI, waist circumference (WC) (23), and insulin resistance (24). Serine (Ser) and Asn are inversely linked to insulin resistance (24). Ornithine (Orn), His, and Pro are positively correlated with an adverse cardiometabolic risk profile in cross-sectional analyses. In addition, Pro and His have been positively associated with insulin resistance (24). Ntzouvani et al. (14) investigated potential patterns in amino acid plasma concentrations using principal component analysis (PCA). They discovered that MetS participants had significantly higher levels of BCAAs, AAAs, Glu, Asp, and Ala, and these amino acid patterns were significantly linked to MetS.

Tau is a sulfur-containing amino acid widely distributed in many tissues and organs. It is involved in various physiological processes. Many studies revealed that Tau effectively reduces cholesterol, triglycerides, blood glucose, and blood pressure (25–28). In a clinical trial with non-diabetic men, who were overweight or obese, oral Tau ameliorates lipid-induced functional β-cell decompensation and insulin resistance by reducing oxidative stress (29). Wu et al. (30) demonstrated that ameliorated hepatic insulin resistance by Tau might be associated with the inhibiting c-Jun N-terminal kinase (JNK1) activation and improving insulin signaling in the liver.

Although studies in other countries have revealed a correlation between amino acids and MetS, few studies existed involving the Chinese Han people. Accordingly, this study discussed the correlation between amino acid profile and MetS and its components in Chinese Han people.

## Materials and Methods

### Research Subjects and Groups

Our study enrolled participants through community-based survey of MetS prevalence. Inclusion criteria include the following: residents from a community, aged 40–65 years, part of the Han population, and have not been intervened with MetS-related components previously. Exclusion criteria include the following: a history of previous cardiovascular events, the use of oral/intravenous glucocorticoids, liver cirrhosis and ascites, kidney damage, hyperthyroidism or hypothyroidism, malignancies, pregnant, or lactating women. We conducted physical examinations, biochemical examinations, and oral glucose tolerance tests. A total of 473 residents were surveyed, and the inclusion criteria for MetS followed the 2009 guidelines of the International Diabetes Federation (IDF) and the American Heart Association/National Heart, Lung, and Blood Institute (AHA/NHLBI) (31). We divided participants into MetS and non-MetS groups and compared the amino acid levels between the two groups.

### Diagnostic Criteria

MetS was defined according to the 2009 guidelines of the IDF and AHA/NHLBI, which include three of the following five items: 1) elevated waist circumference: population and country-specific, a waist circumference of ≥90 cm (Chinese men) or ≥80 cm (Chinese women); 2) elevated triglycerides (or drug treatment for elevated triglycerides) ≥150 mg/dl (1.7 mmol/L); 3) reduced HDL-c (or drug treatment for reduced HDL-c) <40 mg/dl (1.0 mmol/L) in men and <50 mg/dl (1.3 mmol/L) in women; 4) elevated blood pressure (or antihypertensive drug treatment in a patient with a history of hypertension): systolic ≥130 mmHg and/or diastolic ≥85 mmHg; and 5) elevated fasting glucose (or drug treatment of elevated glucose) ≥100 mg/dl (5.6 mmol/L).

### Detection Method

Participants who were not diagnosed with diabetes received a 75-g oral glucose tolerance test (OGTT), whereas those who were previously diagnosed with diabetes were administered a 100-g carbohydrate (steamed bread meal) test. Venous blood samples were obtained at 0 and 2 h following either OGTT or steamed bread meal test. Following standard blood processing, serum and plasma aliquots were stored at −80°C until subsequent use. Using Abbott C16000 automatic biochemical analyzer (Chicago, IL, USA), several biochemical tests were analyzed, including fasting blood glucose (FBG), triglyceride (TG), total cholesterol (TC), low-density lipoprotein-cholesterol (LDL-c), HDL-c, alanine aminotransferase (ALT), aspartate aminotransferase (AST), serum creatinine (CREA), urea nitrogen (BUN), uric acid (UA), and urine albumin-to-creatinine ratio (UACR). Glycosylated hemoglobin (HbA1c) was detected using high-performance liquid chromatography (Hemoglobin Testing System; Bio-Rad, CA, USA). Serum levels of insulin were measured by radioimmunoassay using an insulin detection kit (Beijing North Institute of Biological Technology, China). The homeostatic model assessment of insulin resistance (HOMA-IR) value was employed to evaluate the level of insulin sensitivity and was calculated as follows: fasting blood glucose (mmol/L) × fasting serum insulin (FINS; mU/L)/22.5 (32). Plasma concentrations of 28 amino acids were identified using LC/MS with isotope internal standard method by Beijing Emino Medical Research (Beijing, China). This method is accurate, reliable, and highly reproducible (33, 34). The main instruments used were as follows: the liquid phase model was an HPLC Ultimate3000 (Dionex Liquid Factory, CA, USA), and the mass spectrometer model was a 3200 Q TRAP LC-MS/MS (AB Company, CA, USA).

All participants underwent physical examinations by physicians using standard procedures, including taking measurements of their height, weight, WC, hip circumference (HC), and blood pressure. Blood pressure was measured three times (2 min between each measurement), and the average value was calculated. BMI was calculated by dividing body weight by height squared. Body fat percentage (Fat%) was measured using bioelectrical impedance analysis (TBF-300, Tanita Co., Tokyo, Japan). MRI scans were performed at the umbilicus level between L4 and L5 with the subject in a supine position. The abdominal visceral fat area (VFA) and abdominal subcutaneous fat area (SFA) were calculated using the SliceOmatic software (version 4.2). Smokers were defined as those who smoked at least one cigarette per day during the past year or recently stopped smoking (within the last 12 months); the remaining participants were defined as non-smokers. Alcohol drinkers were defined as those who consumed alcohol more than 3 days a week. Moderate exercise was defined as 30 min of exercise each time, 3–5 days a week.

All participants were interviewed face-to-face by trained medical staff to collect demographic data, baseline lifestyle, and health status data using a standardized questionnaire. This study was approved by the Ethics Committee of Sir Run Run Shaw Hospital. Written informed consent was obtained from all participants.

### Statistical Methods

Data were analyzed using SPSS 26.0 (SPSS Inc., Chicago, IL, USA) and GraphPad Prism (version 7.0; GraphPad Software, Inc.). Categorical variables are expressed as frequencies [*n* (%)]. All continuous variables are reported as mean ± standard deviation (SD), while not normally distributed variables are expressed as median value (interquartile range). The Student’s *t*-test was used to estimate differences in the distribution of demographic characteristics between case and control subjects for continuous variables. The Wilcoxon–Mann–Whitney *U* test was used for not normally distributed variables. One-way analysis of variance was used for intergroup comparisons of MetS components. The association between MetS and categorical variables was evaluated using Fischer’s exact test. A single-factor logistic regression model was employed to assess the correlation between plasma amino acids and MetS to calculate OR value and the corresponding 95% confidence interval, and the difference was statistically significant with *P <*0.05. Following that, logistic regression was deployed to evaluate the link between amino acids and MetS and its components after adjusting for potential covariates, including age, gender, alcohol drinking, current smoking status, and moderate exercise.

Furthermore, Pearson’s correlation coefficients were calculated by using amino acids and metabolic-related variables. The side dendrogram represents the hierarchical clustering of 14 amino acids based on Pearson correlation. Due to the strong correlation between amino acids, PCA extracted the main factors to investigate the link between the amino acid profile and MetS.

## Results

### Population Characteristics

**Table 1** summarizes the clinical and biochemical characteristics of all participants, with an average age of 53.20 ± 6.7 years. The mean age for MetS and non-MetS groups were 56.13 ± 6.40 and 53.15 ± 6.64 years, respectively. Age, BMI, WC, waist-to-hip ratio (WHR), Fat%, SFA, VFA, SBP, DBP, FPG, 2h-PG, FINS, 2h-insulin, HOMA-IR, HbA1c, CHOL, TG, ALT, AST, uric acid (UA), and UACR were higher in the MetS group than in the non-MetS group, but HDL-c was lower. The difference was statistically significant (*P* < 0.05; **Table 1**).

**Table 1:** Comparison of general clinical characteristics and biochemical indicators between the MetS group and non-MetS group.

Variables	Non-MetS group*n* = 401	MetS group*n* = 72	*P*-value
Female, *N* (%)	238 (59.35%)	45 (62.50%)	0.617
Current smoker, *N* (%)	118 (29.35%)	22 (30.56%)	0.982
Alcohol drinker, *N* (%)	163 (40.65%)	32 (44.44%)	0.689
Moderate exercise, *N* (%)	184 (45.77%)	35 (48.61%)	0.655
Age (years)	53.15 ± 6.64	56.13 ± 6.40	<0.001*
BMI (kg/m^2^)	23.04 ± 2.55	26.81 ± 2.58	<0.001*
WC (cm)	77.03 ± 8.11	89.49 ± 6.23	<0.001*
WHR	0.86 ± 0.06	0.93 ± 0.05	<0.001*
Fat% (%)	28.36 ± 6.63	35.06 ± 6.91	<0.001*
SFA (cm^2^)	145.6 (113.05, 192.05)	210 (167.93, 258.30)	<0.001*
VFA (cm^2^)	65.08 (46.21, 103.3)	123.1 (96.06, 151.5)	<0.001*
SBP (mmHg)	120.57 ± 15.35	133.91 ± 15.14	<0.001*
DBP (mmHg)	80.58 ± 13.27	86.00 ± 9.49	0.001*
FPG (mmol/L)	5.02 ± 1.10	5.95 ± 1.81	<0.001*
2h-PG (mmol/L)	6.07 ± 3.14	8.77 ± 4.33	<0.001*
FINS (mIU/L)	10.85 (7.65, 14.16)	16.45 (10.96, 22.88)	<0.001*
2 h insulin (mIU/L)	40.25 (25.86, 62.29)	58.87 (37.86, 100.14)	<0.001*
HOMA-IR	2.37 (1.70, 3.25)	3.66 (2.47, 5.54)	<0.001*
HbA1c (%)	5.67 ± 0.70	6.04 ± 1.00	<0.001*
CHOL (mmol/L)	5.55 ± 1.04	5.86 ± 1.08	0.024*
TG (mmol/L)	1.25 (0.92, 1.65)	1.86 (1.40, 2.60)	<0.001*
LDL-c (mmol/L)	2.41 ± 0.56	2.52 ± 0.63	0.125
HDL-c (mmol/L)	1.48 ± 0.37	1.31 ± 0.31	<0.001*
ALT (IU/L)	18 (14, 26)	24.5 (19, 35.75)	<0.001*
AST (IU/L)	20 (17, 23)	21 (18, 25)	0.005*
CREA (mg/dl)	0.79 ± 0.15	0.80 ± 0.19	0.557
BUN (mmol/L)	16.50 ± 3.84	16.90 ± 5.13	0.447
UA (mg/dl)	4.67 ± 1.39	5.40 ± 1.31	<0.001*
UACR (mg/mmol)	4.59 (3.11, 6.79)	6.99 (4.59, 17.54)	0.001*

Statistical differences between MetS and no MetS are shown as *P < 0.05.

MetS, metabolic syndrome; BMI, body mass index; WC, waist circumference; WHR, waist-to-hip ratio; Fat%, body fat percentage; SFA, subcutaneous fat area; VFA, visceral fat area; SBP, Systolic blood pressure; DBP, diastolic blood pressure; FPG, fasting blood glucose; 2h-PG, 2-h postprandial glucose; FINS, fasting serum insulin; HOMA-IR, homeostasis model assessment for insulin resistance; HbA1c, glycosylated hemoglobin A1c; TC, total cholesterol; TG, triglyceride; LDL-c, low-density lipoprotein-cholesterol; HDL-c, high-density lipoprotein-cholesterol; ALT, alanine aminotransferase; AST, aspartate aminotransferase; CREA, serum creatinine; BUN, serum urea nitrogen; UA, uric acid; UACR, urine albumin-to-creatinine ratio.

### Altered Plasma Amino Acid Levels in MetS

Plasma levels of Ile, Leu, Val, Tyr, Trp, Phe, Glu, Asp, Ala, His, Met, Asn, and Pro were all significantly higher, whereas Tau was significantly lower in the MetS group than those in the non-MetS group, with a statistically significant difference (*P* < 0.05; **Table 2**). The other 14 amino acids were unrelated to MetS.

**Table 2:** Comparison of amino acid determination results between the MetS group and non-MetS group (unit: μmol/L).

	Non-MetS group*n* = 401	MetS group*n* = 72	*P*-value	OR (95% CI)
Isoleucine (Ile)	54.26 ± 12.87	62.50 ± 17.57	<0.001*	1.040 (1.022–1.058)
Leucine (Leu)	133.03 ± 23.87	150.34 ± 28.49	<0.001*	1.026 (1.016–1.037)
Valine (Val)	187.09 ± 35.53	210.27 ± 45.38	<0.001*	1.015 (1.009–1.022)
Tyrosine (Tyr)	72.68 ± 15.10	81.01 ± 18.72	<0.001*	1.031 (1.015–1.046)
Tryptophan (Trp)	30.63 ± 4.94	33.77 ± 6.09	<0.001*	1.113 (1.061–1.168)
Phenylalanine (Phe)	138.91 ± 42.40	158.88 ± 46.43	<0.001*	1.009 (1.004–1.015)
Glutamic (Glu)	36.18 ± 5.99	39.88 ± 7.13	<0.001*	1.090 (1.049–1.134)
Aspartic (Asp)	6.92 ± 1.74	7.70 ± 1.98	<0.001*	1.243 (1.092–1.415)
Alanine (Ala)	202.92 ± 42.25	233.58 ± 38.49	<0.001*	1.017 (1.011–1.023)
Lysine (Lys)	150.43 ± 33.38	156.95 ± 31.31	0.124	1.006 (0.998–1.013)
Arginine (Arg)	124.23 ± 28.03	130.50 ± 29.44	0.083	1.007 (0.999–1.016)
Histidine (His)	97.20 ± 26.30	109.21 ± 38.16	0.001*	1.013 (1.005–1.022)
Methionine (Met)	44.05 ± 11.16	48.16 ± 13.09	0.008*	1.029 (1.008–1.050)
Threonine (Thr)	83.44 ± 23.23	84.64 ± 21.22	0.684	1.002 (0.992–1.013)
Glycine (Gly)	396.11 ± 86.29	391.84 ± 87.80	0.700	0.999 (0.996–1.002)
Serine (Ser)	101.23 ± 17.70	103.37 ± 19.94	0.354	1.007 (0.993–1.020)
Taurine (Tau)	1.57 ± 0.57	1.41 ± 0.52	0.019*	0.559 (0.345–0.905)
Asparagine (Asn)	49.16 ± 11.18	54.33 ± 14.41	<0.001*	1.034 (1.014–1.054)
Citrulline (Cit)	36.08 ± 9.81	37.22 ± 12.27	0.383	1.011 (0.987–1.035)
Ornithine (Orn)	153.15 ± 49.64	161.69 ± 44.26	0.173	1.003 (0.999–1.008)
Glutamine (Gln)	1065.36 ± 314.14	1073.22 ± 274.96	0.842	1.000 (0.999–1.001)
Cysteine (Cys)	143.39 ± 74.01	152.48 ± 66.94	0.331	1.002 (0.998–1.005)
Homocysteine (tHcy)	4.95 ± 1.12	4.68 ± 1.26	0.064	0.809 (0.648–1.010)
α-Aminobutyric acid	2.04 ± 0.66	2.04 ± 0.77	0.992	0.997 (0.689–1.443)
Hydroxyproline	19.44 ± 7.51	20.80 ± 9.19	0.172	1.022 (0.990–1.054)
1-Methylhistidine	33.23 ± 13.48	35.81 ± 14.79	0.141	1.013 (0.996–1.031)
3-Methylhistidine	1.66 ± 2.08	1.92 ± 2.03	0.329	1.054 (0.947–1.173)
Proline (Pro)	308.82 ± 92.76	336.59 ± 83.17	0.018*	1.003 (1.000–1.005)

Statistical differences between MetS and no MetS are shown as *P < 0.05.

### Altered Plasma Amino Acid Profile With MetS Components

All participants were divided into groups according to the number of MetS components (WC, blood glucose, increased TG, decreased HDL-c, and blood pressure). The groups were as follows: 0-component group, 1-component group, 2-components group, and 3–5-components group. The participant who had 3–5 components belonged to the MetS group. As the number of components increased, plasma levels of Ile, Leu, Val, Tyr, Trp, Phe, Glu, Asp, Ala, His, Met, Asn, and Pro increased progressively, but Tau decreased. Compared with the 0-component group, one-way analysis of variance revealed that the other groups exhibited statistically significant higher plasma levels of Ile, Leu, Val, Tyr, Trp, Phe, Glu, Asp, Ala, His, Met, Asn, and Pro and lower plasma levels of Tau (*P* < 0.05; **Table 3**). The other 14 amino acids were unrelated to MetS components.

**Table 3:** Amino acid association with MetS components.

	0 component	1 component	2 components	3–5 components
	*n* = 124	*n* = 165	*n* = 112	*n* = 72
Ile	50.58 ± 11.60	55.16 ± 12.81**	56.96 ± 13.67***	65.31 ± 17.15***
Leu	125.48 ± 22.54	135.18 ± 23.47***	139.41 ± 24.10***	153.39 ± 28.15***
Val	175.45 ± 34.77	192.22 ± 35.02***	196.96 ± 36.39***	208.67 ± 44.91***
Tyr	68.72 ± 14.92	73.67 ± 13.63**	77.60 ± 17.34***	79.68 ± 17.59***
Trp	29.76 ± 5.41	30.92 ± 4.62	31.64 ± 4.65**	33.75 ± 6.50***
Phe	139.42 ± 47.50	139.33 ± 40.37	140.09 ± 35.97	167.43 ± 49.32***
Glu	34.91 ± 6.40	36.51 ± 5.50*	37.70 ± 5.90***	39.76 ± 7.51***
Asp	6.64 ± 1.78	7.04 ± 1.63	7.03 ± 1.61	7.98 ± 2.29***
Ala	185.69 ± 40.88	209.05 ± 40.20***	216.81 ± 41.42***	236.25 ± 34.73***
His	94.27 ± 26.63	97.23 ± 26.62	102.68 ± 26.98*	107.57 ± 38.77*
Met	42.96 ± 12.09	44.43 ± 10.05	45.77 ± 11.90*	46.89 ± 12.94*
Asn	48.55 ± 12.96	49.12 ± 9.72	51.07 ± 11.56	52.86 ± 14.35*
Pro	304.41 ± 116.67	302.49 ± 75.54	326.38 ± 79.00*	335.00 ± 86.17**
Tau	1.59 ± 0.66	1.57 ± 0.51	1.50 ± 0.51	1.39 ± 0.51**

All participants have been divided into four groups according to MetS components and the 14 amino acids associated with MetS in each group. One-way analysis of variance was used, compared with the 0-component group; the statistical differences of other groups are shown as *P < 0.05, **P < 0.01, and ***P < 0.001.

Ile, isoleucine; Leu, leucine; Val, valine; Tyr, tyrosine; Trp, tryptophan; Phe, phenylalanine; Glu, glutamic; Asp, aspartic; Ala, alanine; His, histidine; Met, methionine; Asn, asparagine; Pro, proline; Tau, taurine.

### Amino Acid Profiles Extracted by PCA Were Associated With MetS and Its Components

**Figure 1:**
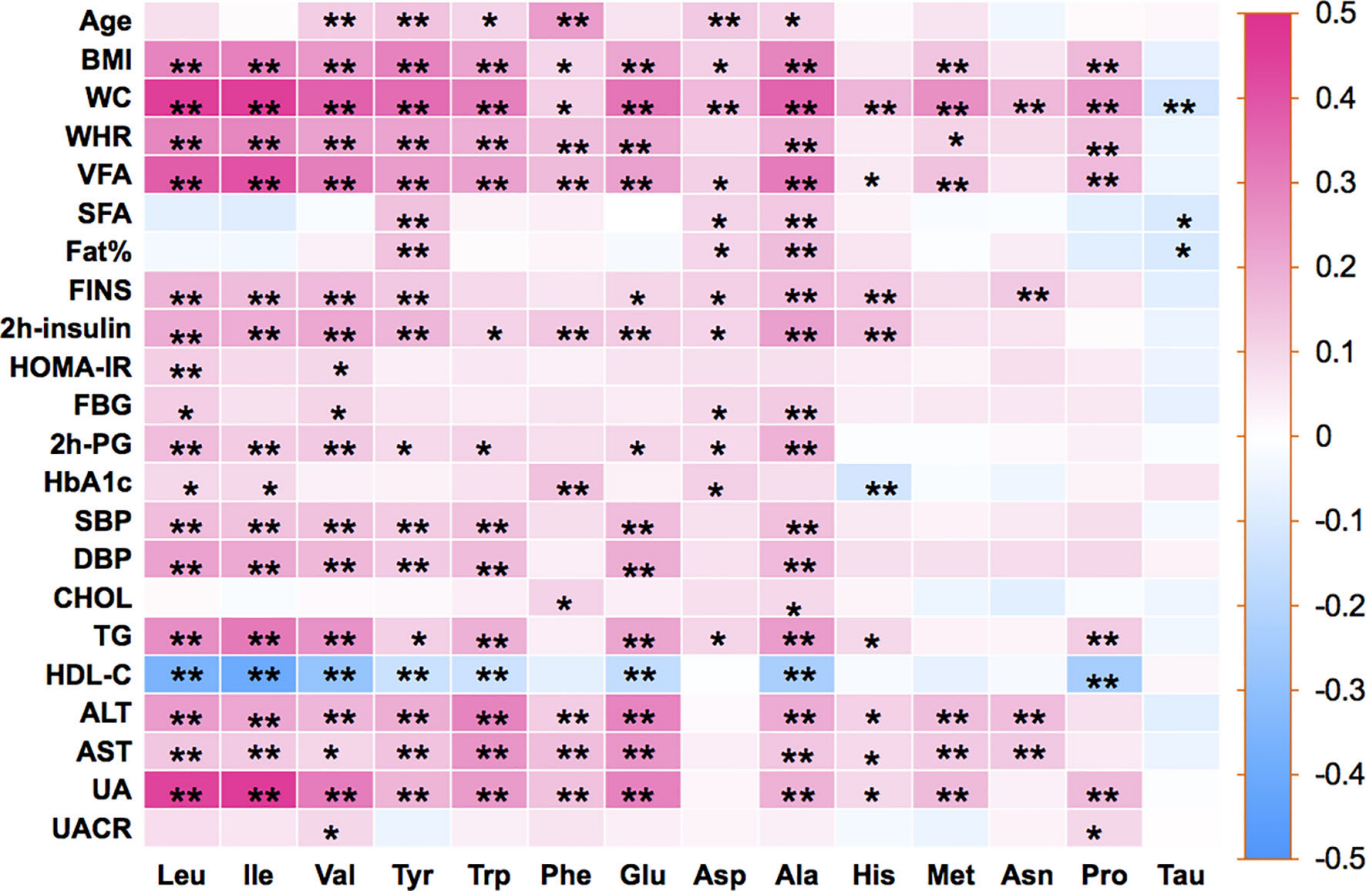
Pearson’s correlation coefficients were calculated between 14 amino acids and metabolic-related variables. BMI, body mass index; WC, waist circumference; WHR, waist-to-hip ratio; Fat%, body fat percentage; VFA, visceral fat area; SFA, subcutaneous fat area; FINS, fasting serum insulin; HOMA-IR, homeostasis model assessment for insulin resistance; FPG, fasting blood glucose; 2h-PG, 2-h postprandial glucose; HbA1c, glycosylated hemoglobin A1c; SBP, systolic blood pressure; DBP, diastolic blood pressure; TC, total cholesterol; TG, triglyceride; HDL-c, high-density lipoprotein-cholesterol; ALT, alanine aminotransferase; AST, aspartate aminotransferase; UA, uric acid; UACR, urine albumin-to-creatinine ratio; Ile, isoleucine; Leu, leucine; Val, valine; Tyr, tyrosine; Trp, tryptophan; Phe, phenylalanine; Glu, glutamic; Asp, aspartic; Ala, alanine; His, histidine; Met, methionine; Asn, asparagine; Pro, proline; Tau, taurine. Statistical differences between 14 amino acids and metabolic-related variables are shown as *P < 0.05 and **P < 0.01.

**Figure 2:**
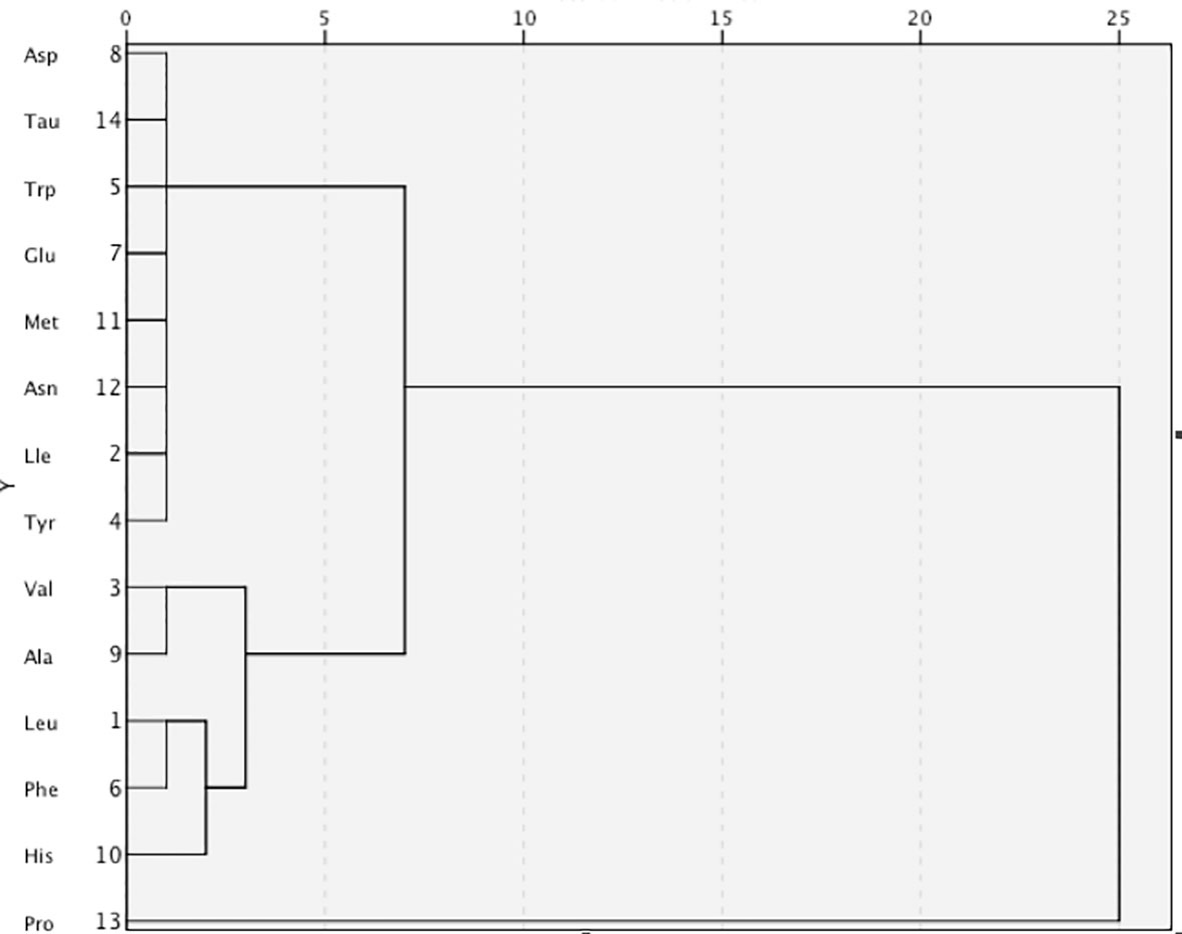
The side dendrogram represents the hierarchical clustering of the 14 amino acids based on Pearson correlation, and we can see a strong correlation with the amino acids. Ile, isoleucine; Leu, leucine; Val, valine; Tyr, tyrosine; Trp, tryptophan; Phe, phenylalanine; Glu, glutamic; Asp, aspartic; Ala, alanine; His, histidine; Met, methionine; Asn, asparagine; Pro, proline; Tau, taurine.

Pearson’s correlation coefficients were calculated between the 14 amino acids and the metabolic variables linked to MetS (**Figure 1**). As illustrated in **Figure 1**, Ile, Leu, Val, Tyr, Trp, Glu, Ala, and Met showed strong positive correlations with BMI, WC, WHR, VFA, insulin, SBP, DBP, TG, and UA, but a strong negative correlation with HDL-c. Only Tau had negative correlations with metabolic variables. The side dendrogram represents the hierarchical clustering of the 14 amino acids based on Pearson correlation, and we can see a strong correlation with the amino acids (**Figure 2**). Multivariate regression analysis cannot be used, and the PCA method was used to investigate their potential patterns. The varimax rotation method was performed to produce interpretable components. Components with an eigenvalue ≥1.0 were extracted. Items with a factor loading ≥│0.4│ were considered as composing a given factor. Factor scores were calculated using the regression method and used in subsequent analyses investigating correlations between extracted factors and MetS components. The amino acid profile, including Ile, Leu, Val, Tyr, Trp, Glu, Asp, Ala, His, Met, Asn, and Pro, denoted “PC1,” was the dominant factor (accounting for 43.29% of the variance in PCA, **Table 4**). This amino acid profile was linked to MetS and its components, related to MetS, abdominal obesity, abnormal glucose, dyslipidemia, and elevated blood pressure after adjustment for age, gender, current smoking, alcohol drinking, and moderate exercise (*P* < 0.05; **Table 5**). Additionally, we identified “PC2”—a Tau-related amino acid profile—as being associated with MetS, abnormal glucose, and dyslipidemia (*P* < 0.05; **Table 5**). PC3 was unrelated to MetS.

**Table 4:** The result from principal component analysis exploring amino acid profiles.

	PC		
	1	2	3
His	**0.545**	**−0.654**	0.027
Ile	**0.761**	0.359	−0.224
Leu	**0.854**	0.321	−0.103
Met	**0.688**	−0.369	−0.075
Phe	0.083	0.373	**0.746**
Trp	**0.815**	0.116	0.115
Val	**0.750**	0.248	−0.166
Tau	−0.066	**0.435**	0.264
Tyr	**0.722**	0.001	−0.105
Asn	**0.675**	**−0.541**	0.181
Asp	**0.616**	−0.124	**0.600**
Glu	**0.828**	0.071	0.036
Ala	**0.701**	0.36	−0.034
Pro	**0.459**	0.295	−0.307
% of variance	43.289	11.438	8.826

Extraction method: principal component analysis (PCA) with varimax rotation; items with a loading ≥│0.4│ were reported as composing a given factor (bold font type).

**Table 5:** The odds ratios for abdominal obesity, abnormal glucose, dyslipidemia, elevated blood pressure, and MetS adjusting for additional factors.

	PC1	*P*-value	PC2	*P*-value
	OR (95% CI)		OR (95% CI)	
Abdominal obesity	1.499 (1.283–1.752)	<0.001***	0.922 (0.776–1.094)	0.352
Abnormal glucose	1.264 (1.077–1.483)	0.004**	1.332 (1.082–1.641)	0.007**
Dyslipidemia	1.339 (1.168–1.535)	<0.001***	1.238 (1.052–1.457)	0.01*
Elevated blood pressure	1.154 (1.011–1.316)	0.033*	0.954 (0.813–1.12)	0.566
MetS	1.723 (1.424–2.085)	<0.001***	1.325 (1.043–1.684)	0.021*

Values are odds ratios (95% confidence intervals) as a continuous variable per SD increment for developing from abdominal obesity, abnormal glucose, dyslipidemia, abnormal blood pressure, and MetS logistic regressions. Adjustment for age, gender, current smoking, alcohol drinking, and physical activity level. Statistical differences between disease and non-disease subjects are shown as *P < 0.05, **P < 0.01, and ***P < 0.001. PC1 (isoleucine, leucine, valine, tryptophan, tyrosine, histidine, methionine, aspartic, asparagine, glutamic, alanine, proline); PC2 (taurine, histidine, asparagine).

MetS, metabolic syndrome; OR, odds ratio.

### Amino Acid Profiles Were Associated With Future Development of MetS Within 5 Years

To confirm the correlation of amino acid profiles and future development of MetS, we followed up 401 non-MetS. Finally, 260 participants were followed up effectively from 2010 until 2015 (another 141 participants were lost to follow-up due to moving to another house, requesting to withdraw from the study, etc.), and 42 participants developed new-onset MetS. The basal clinical and biochemical characteristics were compared according to whether participants developed new-onset MetS or not within 5 years. We found that the baselines of BMI, WC, WHR, Fat%, VFA, SBP, FINS, 2h-insulin, HOMA-IR, TG, and UA were significantly higher in the subsequent new-onset MetS group than in the subsequent non-MetS group, while HDL-c was significantly lower (*P* < 0.05; **Table 6**). Ile, Leu, Tyr, and Ala were elevated, and Tau was reduced in the new-onset MetS group at baseline, exhibiting a statistically significant difference (*P* < 0.05; **Table 7**). After adjusting for age, gender, current smoking, alcohol drinking, and moderate exercise, the “PC1” and Tau were significantly correlated with MetS (*P* < 0.05). After adjusting for BMI, WC, WHR, Fat%, VFA, SBP, FINS, 2h-insulin, TG, HDL-c, and UA, Tau was significantly negatively correlated with MetS (*P* = 0.016). Decreased Tau could be associated with future development of MetS within 5 years.

**Table 6:** Comparison of the baseline of general clinical characteristics and biochemical indicators between 42 subsequent new MetS and 218 subsequent non-MetS.

Variables	Subsequent non-MetS*n* = 218	Subsequent new MetS*n* = 42	*P-*value
Female, *N* (%)	131 (60.09%)	20 (47.62%)	0.198
Current smoker, *N* (%)	48 (22.02%)	11 (26.19%)	0.531
Alcohol drinker, *N* (%)	88 (40.37%)	18 (42.86%)	0.749
Moderate exercise, *N* (%)	107 (49.08%)	17 (40.48%)	0.334
Age (years)	53.01 ± 6.34	53.49 ± 7.46	0.856
BMI (kg/m^2^)	22.44 ± 2.47	25.07 ± 1.96	<0.001*
WC (cm)	75.35 ± 7.71	83.19 ± 6.04	<0.001*
WHR	0.85 ± 0.06	0.90 ± 0.05	<0.001*
Fat% (%)	27.58 ± 6.76	30.95 ± 5.06	0.007*
SFA (cm^2^)	141.3 (110.3, 189.35)	155.3 (126.6, 203.9)	0.112
VFA (cm^2^)	63.66 (36.42, 89.54)	104.5 (66.68, 128.75)	<0.001*
SBP (mmHg)	119.27 ± 14.90	125.50 ± 15.26	0.023*
DBP (mmHg)	80.65 ± 16.08	84.01 ± 9.35	0.268
FPG (mmol/L)	4.95 ± 0.84	5.09 ± 0.97	0.361
2h-PG (mmol/L)	5.91 ± 2.79	6.55 ± 3.39	0.225
FINS (mIU/L)	10.39 (6.87, 13.33)	13.67 (8.61, 18.64)	<0.001*
2 h insulin (mIU/L)	37.25 (24.60, 60.30)	56.29 (32.17, 87.59)	0.034*
HOMA-IR	2.13 (1.49, 3.07)	3.09 (1.99, 4.20)	<0.001*
HbA1c (%)	5.65 ± 0.63	5.61 ± 0.59	0.689
CHOL (mmol/L)	5.52 ± 1.09	5.58 ± 0.85	0.738
TG (mmol/L)	1.19 (0.80, 1.54)	1.62 (1.26, 2.47)	0.002*
LDL-c (mmol/L)	2.41 ± 0.56	2.40 ± 0.53	0.948
HDL-c (mmol/L)	1.51 ± 0.38	1.28 ± 0.34	0.001*
ALT (IU/L)	19 (14, 26)	21 (16, 27.75)	0.657
AST (IU/L)	20 (17, 23)	20 (17.25, 24.75)	0.688
CREA (mg/dl)	0.80 ± 0.16	0.84 ± 0.26	0.709
BUN (mmol/L)	16.76 ± 3.59	16.11 ± 3.55	0.318
UA (mg/dl)	4.58 ± 1.39	5.34 ± 1.84	0.002*
UACR (mg/mmol)	4.43 (2.79, 12.09)	4.59 (3.13, 7.03)	0.15

After a 5-year follow-up, 260 non-MetS were followed up effectively, of which 42 developed new MetS. Statistical differences between subsequent new MetS and subsequent non-MetS are shown as *P < 0.05.

MetS, metabolic syndrome; BMI, body mass index; WC, waist circumference; WHR, waist-to-hip ratio; Fat%, body fat percentage; SFA, subcutaneous fat area; VFA, visceral fat area; SBP, systolic blood pressure; DBP, diastolic blood pressure; FPG, fasting blood glucose; 2h-PG, 2-h postprandial glucose; FINS, fasting serum insulin; HOMA-IR, homeostasis model assessment for insulin resistance; HbA1c, glycosylated hemoglobin A1c; TC, total cholesterol; TG, triglyceride; LDL-c, low-density lipoprotein-cholesterol; HDL-c, high-density lipoprotein-cholesterol; ALT, alanine aminotransferase; AST, aspartate aminotransferase; CREA, serum creatinine; BUN, serum urea nitrogen; UA, uric acid; UACR, urine albumin-to-creatinine ratio.

**Table 7:** The baseline of plasma amino acid concentrations in subsequent new MetS and subsequent non-MetS groups.

	Subsequent non-MetS (n=218)	Subsequent new MetS (n=42)	*P*-value	OR (95% CI)
Leu	130.46 ± 23.15	141.46 ± 21.83	0.009*	1.020 (1.005–1.036)
Ile	53.25 ± 12.83	59.29 ± 11.39	0.01*	1.036 (1.009–1.064)
Val	186.17 ± 35.74	195.97 ± 36.24	0.13	1.008 (0.998–1.017)
Tyr	70.18 ± 13.50	76.20 ± 14.14	0.017*	1.031 (1.015–1.046)
Trp	30.11 ± 5.01	30.80 ± 4.37	0.437	1.028 (0.958–1.104)
Phe	141.11 ± 41.19	149.74 ± 38.17	0.122	0.993 (0.983–1.002)
Glu	35.59 ± 6.06	37.03 ± 5.60	0.182	1.040 (0.982–1.101)
Asp	6.71 ± 1.58	7.15 ± 1.90	0.134	1.169 (0.953–1.433)
Ala	196.27 ± 40.33	211.39 ± 43.24	0.042*	1.009 (1.000–1.018)
His	97.19 ± 26.77	103.88 ± 24.99	0.162	1.009 (0.996–1.022)
Met	42.89 ± 9.76	46.25 ± 11.03	0.063	1.033 (0.998–1.069)
Asn	48.63 ± 11.02	50.74 ± 8.81	0.272	1.018 (0.986–1.051)
Pro	300.04 ± 94.72	309.83 ± 65.48	0.551	1.001 (0.998–1.004)
Tau	1.68 ± 0.59	1.37 ± 0.53	0.003*	0.325 (0.153–0.686)

Statistical differences between subsequent new MetS and subsequent non-MetS are shown as *P < 0.05.

Ile, isoleucine; Leu, leucine; Val, valine; Tyr, tyrosine; Trp, tryptophan; Phe, phenylalanine; Glu, glutamic; Asp, aspartic; Ala, alanine; His, histidine; Met, methionine; Asn, asparagine; Pro, proline; Tau, taurine.

## Discussion

Using the 2009 diagnostic criteria of the IDF and AHA/NHLBI, we examined the association between amino acids and MetS. First, we found evidence that amino acid profiles are beneficial in identifying individuals who are at high risk of MetS in a Chinese Han population. It has been demonstrated that increased levels of Ile, Leu, Val, Tyr, Trp, Phe, Glu, Asp, Ala, His, Met, Asn, and Pro and decreased Tau levels were linked to an increased risk of MetS and its components.

As revealed in this study, we have stated that BCAAs, AAAs, Glu, and Ala were positively associated with BMI, WC, WHR, VFA, FINS, 2h-insulin, 2h-PG, SBP, DBP, TG, and UA and negatively associated with HDL-c. Our study also discovered that His was positively associated with WC and insulin resistance. Met and Pro were positively linked to BMI, WC, and VFA. Gly and Cys did not differ significantly in the Chinese Han population in this study. However, we discovered that Tau was significantly lower in MetS and was negatively correlated with metabolic-related variables. Numerous studies with rats, mice, and rabbits revealed that Tau effectively reduces TC, TG, blood glucose, and blood pressure (35–39).

While interethnic differences in the pathophysiology of insulin dysregulation and visceral obesity between Asian and Western populations are well known, few studies have examined the relationship between amino acids and lifestyle-related disease risks in Chinese populations (40). Our research has extracted “PC1” using PCA. It was the dominant factor (accounting for 43.29% of the variance in PCA) and was mostly constituted of BCAAs, Tyr, Trp, Glu, Asp, Asn, Ala, His, Met, and Pro. After adjusting for age, gender, smoking, drinking, and moderate exercise, the amino acid profile of PC1 was linked to abdominal obesity, abnormal glucose, dyslipidemia, elevated blood pressure, and MetS. After a 5-year follow-up, this amino acid profile remained significantly correlated with the future incidence of MetS within 5 years. BCAAs and AAAs (except Phe) exhibited the highest loadings in PC1, consistent with previous research from other countries indicating that BCAA/AAA pattern was connected with MetS and its components. However, the correlation between Phe and MetS is not strong in Chinese Han populations. Yamakado et al. (11) used a method other than PCA to calculate a plasma free amino acid (PFAA) index but also discovered that indices that included BCAAs and AAAs were positively associated with VFA and circulating insulin levels in cross-sectional analyses, confirming that plasma free amino acid profiles could predict the future development of DM, MetS, and dyslipidemia in the Japanese population, even within a relatively short period (4 years). Other studies performed a systems metabolomics approach to predict MetS development, but these predictive models are specific to the T2DM component of MetS. Therefore, the prediction of MetS subjects without a T2DM component reveals a high rate of misclassified subjects (around 30%), implying poor prediction capacity of MetS hypertension and dyslipidemia components (41). Our amino acid profile was associated with MetS and its components (abdominal obesity, abnormal glucose, dyslipidemia, and elevated blood pressure). It can be employed as a potential biomarker for assessing MetS risk.

The amino acid profile, which mainly included Tau, His, and Asn, denoted “PC2,” accounted for 11.44% of PCA variance. This amino acid profile was associated with abnormal glucose, dyslipidemia, and MetS after adjustment for age, gender, smoking, drinking, and moderate exercise. After a 5-year follow-up, PC2 exhibited no significant difference with future development of MetS. However, Tau was significantly negatively correlated with MetS after adjusting for age, gender, smoking, drinking, exercise, BMI, WC, Fat%, VFA, SBP, FINS, TG, HDL-c, and UA. Decreased Tau could be linked with the future development of MetS within 5 years. Other investigations have demonstrated that patients with obesity (42) and diabetes (43) have lower Tau levels in their bodies, consistent with our finding that Tau decreased in MetS. The decrease in blood Tau levels was associated with a decrease in cysteine dioxygenase expression, a rate-limiting enzyme in taurine synthesis (42). In contrast, Tau supplementation increases plasma Tau levels, reduces plasma levels of inflammatory and oxidative markers, and increases plasma adiponectin levels in humans. Research studies have indicated that Tau prevents obesity mainly due to increasing energy expenditure by upregulating relative factor expression involved in fatty acid oxidation. It also prevents hypercholesterolemia by promoting bioconversion of cholesterol to bile acids, promoting bile acid excretion in feces, and suppressing bile acid absorption from the ileum. It also prevents diabetes mellitus by exerting antioxidant and anti-inflammatory effects, protecting pancreatic β cells, promoting insulin secretion, improving insulin resistance by inhibiting JNK1 activation, improving insulin signaling in the liver, and preventing hypertension by suppressing RAAS. Tau may be beneficial for preventing MetS (30, 44, 45).

## Conclusion

This cross-sectional study of a cohort investigated the correlation between amino acids and MetS in a Chinese Han population. In the present study, we extracted the amino acid profile using PCA, which can be used as biomarkers to assess and monitor MetS risk. Tau may be beneficial for preventing MetS. Decreased Tau levels were associated with the future development of MetS. However, this study has shortcomings, such as that it only involved participants aged 40–65 years old and the number of samples with clinical follow-up was small, implying that more samples are required to verify these results.

## Data Availability Statement

The original contributions presented in the study are included in the article/supplementary material. Further inquiries can be directed to the corresponding authors.

## Ethics Statement

Written informed consent was obtained from the individual(s) for the publication of any potentially identifiable images or data included in this article.

## Author Contributions

All authors listed have made a substantial, direct, and intellectual contribution to the work and approved it for publication.

## Funding

This work was supported by the Program for Zhejiang Leading Team of Science and Technology Innovation (2012R10050-03).

## Conflict of Interest

The authors declare that the research was conducted in the absence of any commercial or financial relationships that could be construed as a potential conflict of interest.

## Publisher’s Note

All claims expressed in this article are solely those of the authors and do not necessarily represent those of their affiliated organizations, or those of the publisher, the editors and the reviewers. Any product that may be evaluated in this article, or claim that may be made by its manufacturer, is not guaranteed or endorsed by the publisher.
